# A Screen for Genes Expressed in the Olfactory Organs of *Drosophila melanogaster* Identifies Genes Involved in Olfactory Behaviour

**DOI:** 10.1371/journal.pone.0035641

**Published:** 2012-04-18

**Authors:** Narelle E. Tunstall, Anabel Herr, Marien de Bruyne, Coral G. Warr

**Affiliations:** School of Biological Sciences, Monash University, Clayton, Victoria, Australia; University of Houston, United States of America

## Abstract

**Background:**

For insects the sense of smell and associated olfactory-driven behaviours are essential for survival. Insects detect odorants with families of olfactory receptor proteins that are very different to those of mammals, and there are likely to be other unique genes and genetic pathways involved in the function and development of the insect olfactory system.

**Methodology/Principal Findings:**

We have performed a genetic screen of a set of 505 *Drosophila melanogaster* gene trap insertion lines to identify novel genes expressed in the adult olfactory organs. We identified 16 lines with expression in the olfactory organs, many of which exhibited expression of the trapped genes in olfactory receptor neurons. Phenotypic analysis showed that six of the lines have decreased olfactory responses in a behavioural assay, and for one of these we showed that precise excision of the P element reverts the phenotype to wild type, confirming a role for the trapped gene in olfaction. To confirm the identity of the genes trapped in the lines we performed molecular analysis of some of the insertion sites. While for many lines the reported insertion sites were correct, we also demonstrated that for a number of lines the reported location of the element was incorrect, and in three lines there were in fact two pGT element insertions.

**Conclusions/Significance:**

We identified 16 new genes expressed in the *Drosophila* olfactory organs, the majority in neurons, and for several of the gene trap lines demonstrated a defect in olfactory-driven behaviour. Further characterisation of these genes and their roles in olfactory system function and development will increase our understanding of how the insect olfactory system has evolved to perform the same essential function to that of mammals, but using very different molecular genetic mechanisms.

## Introduction

The olfactory systems of insects allow them to recognise and discriminate amongst a large number of odorants, vital for finding food resources, oviposition sites, and identifying mates. *Drosophila melanogaster* offers distinct advantages as a model system for studying olfaction. As in other insects, electrophysiological recording techniques allow response properties of single olfactory neurons (mostly determined by single odorant receptors) to be measured with relative ease. Olfactory-driven behaviours can be measured using laboratory-based behavioural assays. Finally, *Drosophila* has the additional advantages of powerful molecular genetic techniques for studying gene expression and function.

In *Drosophila*, odours are detected by different functional classes of olfactory receptor neurons (ORNs), located in two pairs of olfactory organs, the third antennal segments and the maxillary palps. Individual ORNs respond to multiple odorants, and most odorants are detected by multiple classes of ORN, though typically with different sensitivities [1], [2]. The responses of most insect ORNs are reliant on members of two insect-specific families of olfactory receptor proteins. The largest and best characterised family of olfactory receptors, encoded by the *Or* genes [3], [4], have an inverted membrane topology compared to mammalian receptors [5], [6], and do not primarily signal through G-proteins [6], [7]. Instead it appears that the insect Or proteins form a novel class of heteromeric cation channels, directly gated by odorants [7], [8]. The functional receptor is believed to be a multimer comprising at least one variable Or odorant-binding subunit and at least one copy of a co-receptor subunit called Orco [5], [7], [8]. In addition, a second family of olfactory receptors was recently discovered in *Drosophila*, encoded by the *IR* gene family. These genes also encode ion channels, in this case related to ionotropic glutamate receptors [9].

Extensive studies have been performed over the past decade to elucidate the roles of the odorant receptors in *Drosophila* olfaction. However, aside from the large family of odorant binding proteins, whose functions are still unclear, relatively few other genes involved in olfactory system function and development have been identified. In an effort to identify novel genes important for olfactory system function or development here we conducted a screen to identify genes expressed in the adult olfactory organs. We screened a set of p{GT1} “gene trap” *P* element insertion lines (hereafter abbreviated as “pGT”) generated by the Berkeley *Drosophila* Genome Project (BDGP) Gene Disruption project [10].

The pGT lines contain insertions of a *P*-element vector designed to enable selection of inserts within the transcription units of genes, rather than upstream of genes as often occurs with conventional enhancer trap lines [11]. The aim is thus to generate a hypomorphic mutation in addition to reporting gene expression, and to achieve this aim the pGT1 vector contains two key marker genes. The *Gal4* gene in the vector lacks a promoter but has a poly(A)^+^ signal sequence, and is preceded by an artificial splice acceptor site. This allows the *Gal4* sequence to be transcribed as a fusion mRNA with an upstream exon sequence. It will not be expressed unless the vector integrates downstream of the promoter of a host gene, whereupon *Gal4* expression will reflect the expression pattern of the host gene. The *mini-w* gene in the vector has a promoter but lacks a poly(A)^+^ signal sequence, and is followed by an artificial splice donor site. This allows the *mini-w* sequence to be transcribed as part of a chimaeric mRNA with exonic sequence of a host gene 3′ to the insertion site. The *mini-w* mRNA will only be polyadenylated if it is spliced to a host gene exon, thus enabling the identification of lines containing insertions within transcription units of genes by selecting for red eye colour. The BDGP group used this latter feature to generate a set of pGT lines and then mapped their insertion sites in the genome by using inverse PCR to determine the genomic sequences flanking the insertion sites. Thus candidate trapped genes for lines of interest can be identified from the Flybase database [12].

We utilised the enhancer trap feature of the pGT element to screen 505 available lines for expression in the *Drosophila* olfactory organs. For those lines in which we observed olfactory tissue expression we then carried out where possible both phenotypic analysis to look for olfactory defects, as well as molecular analysis to confirm the identity of the trapped genes. We identified 16 lines with expression in the olfactory organs, only one of which appeared to be olfactory-specific. Molecular analysis of some of the insertion sites demonstrated that in a number of lines the reported location of the element was incorrect, and in three lines there were in fact two pGT element insertions. Olfactory behaviour assays were carried out for seven of the olfactory-expressed lines that were homozygous viable, and six were found to have defects in olfactory-driven behaviour. For two lines with severe olfactory behaviour defects we precisely excised the P element and showed that the olfactory behaviour defect is caused by the P element insertion for one, confirming that the gene trapped in this line is required for olfactory-driven behaviour.

## Results

### A subset of pGT lines are expressed in olfactory organs

505 pGT lines were screened for expression in the olfactory organs of adult flies by crossing them to the reporter line UAS-mCD8:GFP [13]. GFP expression patterns of progeny containing both transgenes were observed through the cuticle and documented for the olfactory organs. 16 lines were positive for GFP expression in the third antennal segment (Table 1). In all but two of these lines expression was also seen in the maxillary palps (Table 1). Expression in the maxillary palps alone was not observed in any of the lines screened. We did not find clear evidence for expression in particular morphological types of sensilla in any of the lines. For these 16 lines we then examined expression in other sensory tissues (mouthparts, second antennal segment, legs, wings) using whole flies, and in the brain using head cryo-sections. Only one line (BG01140) showed specific expression in the olfactory organs only and was not detected elsewhere. All of the remaining lines had additional GFP expression in various tissues, and interestingly in many cases these were other sensory tissues (Table 1).

**Table 1:** GFP expression patterns observed in ‘olfactory positive’ pGT lines.

Line	Third antennal segment	Maxillary Palp	Mouth	Second antennal segment	Leg	Wing	Brain	Cellular location
BG00076	+	−	−	+	+	+	+	Neurons
BG00842	+	+	−	−	−	−	−	Accessory cells
BG00973	+	+	+	+	+	−	−	Neurons^a^
BG01140	+	+	−	−	−	−	−	Accessory cells
BG01171	+	+	−	+	+	−	+	Neurons
BG01322	+	−	+	+	+	+	+	Neurons^a^
BG01610	+	+	+	+	+	+	+	Neurons
BG01711	+	+	+	+	+	+	+	Neurons
BG01746	+	+	+	+	+	+	+	Neurons^a^
BG02142	+	+	+	+	+	−	+	Neurons^a^
BG02184	+	+	+	−	−	−	−	Accessory cells
BG02427	+	+	+	+	+	−	+	Neurons
BG02759	+	+	+	+	+	+	+	Neurons and accessory cells
BG02810	+	+	+	+	+	+	+	Neurons
BG02820	+	+	+	+	+	−	+	Neurons
BG02836	+	+	+	+	+	−	+	Neurons^a^

*Note*. Mouth - Mouthparts including proboscis, labellum and/or cibarial organs. Leg - Tips/distal parts or joints of legs. Wing - Wing margin or joints of wings. Brain - Majority of lines had staining in the mushroom bodies or uniformly in the brain, some lines also had staining in the optic lobes.

a These lines showed inconsistent Elav co-localisation patterns and expression is also possibly in accessory cells.

**Figure 1:**
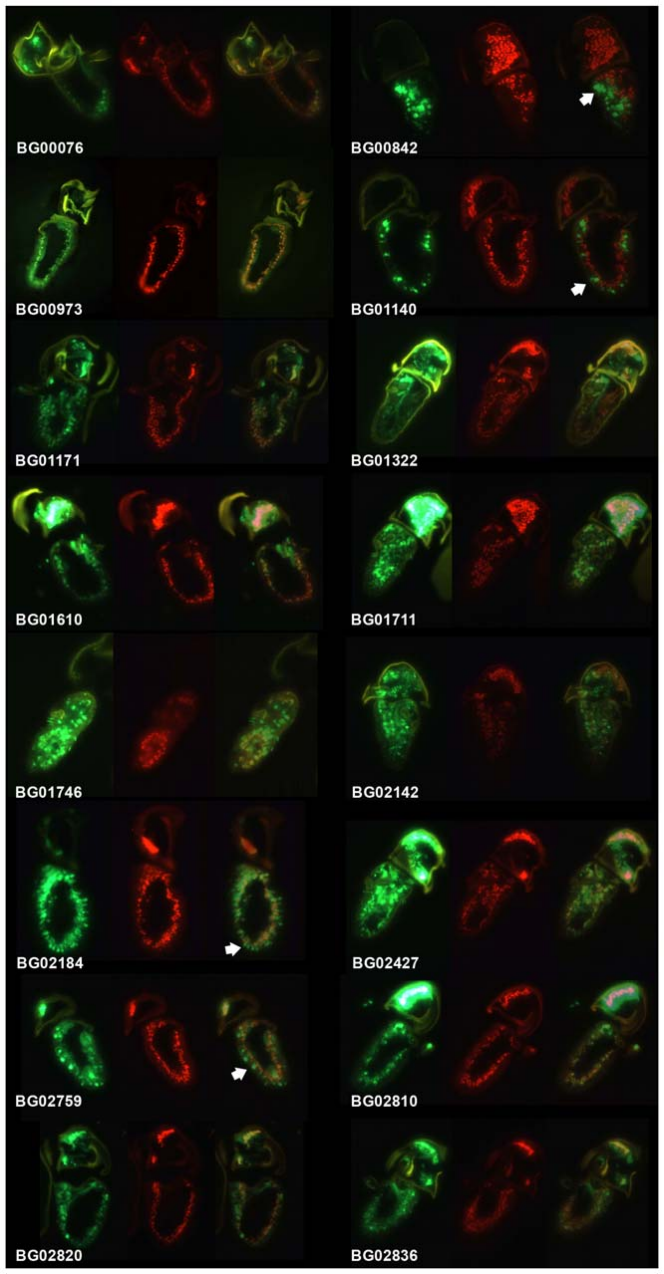
GFP expression patterns of pGT lines in olfactory organs. Cryostatic antennal sections were double stained with α-GFP (green) and α-ELAV (red) and then the images overlain. The GFP reporter used is mcd8:GFP which localises to membranes. For most lines expression is neuronal as indicated by co-localization of GFP and ELAV. Note that when expressed in olfactory neurons mcd8:GFP is localised to membranes of cell bodies encircling ELAV staining of the cell nucleus, and also extends into dendrites (sensillum shafts) and axons. In BG00842, BG01140 and BG02759 α-GFP labels larger cells at the base of sensilla while α-ELAV labels the neuronal nuclei located more deeply, indicating the expression of GFP is in accessory cells. In BG02184 expression is seen in both neurons and accessory cells. Examples of cells showing GFP fluorescence but negative for ELAV are indicated with arrows. These sections are representative of 10–20 examined for each line.

We then asked whether the GFP expression in the olfactory organs was neuronal or due to expression in another cell type (such as accessory cells). By examining GFP fluorescence in antennae and maxillary palps on head cryo-sections we found that different lines showed obvious differences in cellular expression patterns. In some lines expression in antennae and palps appeared neuronal, in others it was more suggestive of accessory cells. To confirm if expression was neuronal we performed co-localisation studies with the Elav pan-neuronal marker for the 16 lines. 12 lines clearly showed GFP co-localisation with Elav, confirming expression in neurons (Figure 1; Table 1), however in three lines it did not co-localise. In these lines GFP expression was localised to larger cells at the base of the sensillum that did not appear to have a dendritic process, whereas neuronal nuclei are located further away from the cuticle (Figure 1; Table 1). This strongly suggests expression in the accessory cells which are wrapped around the apical aspect of the olfactory neurons. In the remaining line (BG02759), GFP appeared to be localised to both neurons and accessory cells (Figure 1).

### Identification of trapped genes

What genes have had their expression pattern trapped in these 16 lines? The BDGP used inverse PCR to map the pGT vector insertion sites in the genome. While in some of the lines we could use this location to determine a candidate for the trapped gene, at the time we conducted the screen the Flybase annotation for a number of the lines showed the insertion sites in locations nowhere near a predicted gene. Due to this we performed experiments, described below, to locate the inserts in some of the lines. In the latest *Drosophila* genome annotation release, however, annotation of new isoforms of many genes has occurred (due to new RNA sequencing data) and shows that some of the pGT insertions for which we could not initially find a candidate gene are in fact located within previously unannotated introns (often quite large). Thus for all but one of the lines (BG00973) we can now assign a candidate gene based on the BDGP-determined insertion site (Table 2). However, our analysis did uncover some discrepancies, in several cases a different insertion site for the element to that determined by BDGP was identified, in others we identified the presence of two insertion sites.

**Table 2:** Molecular analysis of pGT lines and predicted candidate genes.

Line	No. of inserts^a^	BDGP predicted gene^b^	Cyt.^c^	3′ RACE gene^d^	Cyt.^e^	Candidate Gene	Insert position	Predicted Function/Structural Domains
BG00076	1	*SKIP*	94A2	N.D.		*SKIP*	5′UTR	Shal-interacting protein, SH2-SH3 domains
BG00842	1	*CG15095*	55F4	N.D.		*CG15095*	2.6 kb 5′ to CG15095	Na-P symporter
BG00973	2	None	61D1	*CG3217*	61C8	*CG3217*	Intron	Unknown
BG01140	1	*MYPT-75D*	75D5	*Mctp*	55F1-2^f^	*Mctp*	3′ UTR	Multiple C2 transmembrane protein
BG01171	2	*Aef1*	78D2	N.D.		*Aef1* g	Exon	C2H2 Zn finger transcription factor
BG01322	1	*shep*	64C9	*shep*	64C9	*shep*	Intron	mRNA binding protein
BG01610	1	*sbb*	55C2	N.D.		*sbb*	Intron	C2H2 Zn finger transcription factor
BG01711	1	*fz2*	76A1	*CG8771*	49B12	*fz2* or *CG8771*	Intron	*fz2*: Wnt receptor, *CG8771*: Unknown
BG01746	1	*tai*	30A4	N.D.		*tai*	Intron	Co-activator of ecdysone receptor
BG02142	1	*Pka-C1*	30C5	N.D.		*Pka-C1*	5′ UTR	cAMP dependent Ser/Thr kinase
BG02184	1	*CAP* or *CG12309*	46F9 or 47B7	*CAP*	46F9	*CAP*	Intron	Vinculin binding, cell adhesion
BG02427	1	*CG42669*	62E1	*CG42699*	5C2	*CG42669* or *CG42699*	Intron	Unknown for both genes
BG02759	1	*Pdk1*	61B1	N.D.		*Pdk1*	Intron	Phosphoinositide-dependent kinase 1
BG02810	1	*cpo*	90D1	N.D.		*cpo*	Intron	mRNA binding protein
BG02820	3	*chif* or *CG42231*	35F12	N.D.		*chif* or *CG42231*	Intron shared by both genes	*chiffon*: Zn finger DNA-binding protein, *CG42231*: unknown
BG02836	1^h^	*Pfrx*	18C8	*A2BP1*	67E4	*Pfrx* or *A2BP1* h	Intron	*Pfrx*: 6-phosphofructo-2-kinase, *A2BP1*: RNA binding-protein/splicing regulator

*Note*.

a Number of pGT inserts identified by Southern blot analysis.

b BDGP prediction as taken from Flybase.

c Cytological location of BDGP predicted gene.

d Candidate gene identified from 3′RACE experiments.

e Cytological location of gene identified by RACE.

f Cytological location as determined by polytene chromosome *in situ* hybridisation with a *Gal4* probe.

g As the Southern blot indicates two inserts there may be a second unidentified candidate gene for this line.

h For BG02836 a Southern blot suggested one insert but polytene chromosome *in situ* hybridisation gave two signals, one at ∼18D1 and one at ∼67E thus there may be two inserts. N.D. – not determined.

We performed a number of experiments to determine which gene the vector was being spliced to in the lines. First, we investigated the possibility that a reason the BDGP mapped insertion sites could in some cases appear to be large distances from any annotated genes could be the presence of multiple insertion sites, one being the insertion site mapped by BDGP and a second being the insertion site that has trapped the gene of interest. In order to determine the number of inserts in each line we performed Southern blots on genomic DNA from each line and probed for the *Gal4* gene present in the pGT construct. This showed that while most lines had a single pGT insertion, three had multiple insertions; BG00973 and BG01171 have two, and BG02820 has three (Table 2; Figure S1). In these three lines any of the insertions could thus be producing the olfactory expression pattern.

We then performed 3′ RACE to identify the exons to which the pGT vector sequences had spliced. A pGT element insertion is predicted to produce two transcripts, one containing a 5′ exon of the trapped gene spliced to an exon encoding the *Gal4* gene, and a second containing an exon encoding the *mini-white* gene spliced to a 3′ exon of the trapped gene [11]. We used a primer designed to the *white* gene in 3′RACE experiments, and 3′RACE products were successfully obtained from seven of the lines (Table 2).

The RACE results confirmed that the vector splices to the BDGP-identified genes in two of these seven lines, BG01322 and BG02184. These lines were thus confirmed to trap the genes *shep* and *CAP* respectively.

For the line BG00973, BDGP had mapped the insert to a location with no annotated genes within 20 kb in either direction and thus there was no predicted gene. We found that the vector was spliced to the gene *CG3217*, which is ∼70 Kb upstream from the BDGP-mapped position. Southern blots had indicated the presence of two insertions in this line, and using PCR with primers located within the P element sequence and in predicted flanking genomic DNA we confirmed this to be the case. One insertion is at the site predicted by BDGP, a second is in the 3rd intron of *CG3217* (between exons 3 and 4). Thus *CG3217* is the candidate olfactory-expressed gene for this line.

For the other four lines, BG01140, BG01711, BG02427 and BG02836, we identified different candidate genes to those predicted by BDGP (Table 2).

For BG01140, the RACE results showed the vector was spliced to the *Mctp* gene at cytological region 55F, contradicting the BDGP prediction at 75F in the *MYPT-75D* gene. This was thus further investigated by performing polytene chromosome DNA *in situ* hybridisations using a probe for the *Gal4* gene. A signal at 55F was observed (Figure S2), which fits our 3′RACE predicted gene *Mctp* and not that identified by BDGP. Thus *Mctp* is the candidate olfactory-expressed gene for this line.

For BG02836, the RACE results showed the vector was spliced to the *A2BP1* gene at cytological region 67E4, contradicting the BDGP prediction at 18C8 in the *Pfrx* gene. Our Southern blot experiment had suggested only one insert, however two signals were observed in our polytene chromosome *in situ* hybridisation, one at ∼18D1 and one at ∼67E (Figure S2). This suggests there are two inserts in this line, this could appear as only one band on a Southern blot if both insert locations produce similarly sized restriction fragments. Thus for BG02836 both the *A2BP1* and *Pfrx* genes are candidates.

For BG01711, the RACE results showed the vector was spliced to the *CG8771* gene at cytological region 49B12, contradicting the BDGP prediction at 76A1 in the *Frizzled 2* gene. For BG02427, the RACE results showed the vector was spliced to the *CG42699* gene at cytological region 5C2, contradicting the BDGP prediction at 62E1 in the *CG42669* gene. For both these lines we were unable to obtain polytene chromosome *in situ* results and thus for each line both genes remain candidates (Table 2).

By combining the 3′RACE and polytene chromosome *in situ* data with the BDGP data we thus determined the most likely gene(s) trapped in each of the 16 lines, as listed in Table 2.

### Olfactory behaviour defects were observed for six pGT lines

We next wanted to determine if the insertion of the pGT element into olfactory-expressed genes in the lines caused any defects in olfactory behaviour due to hypomorphic mutations. We were interested in genes affecting the detection or processing of olfactory information but found that the pGT insertion appeared to affect viability in nine of the lines suggesting effects on more essential functions. Homozygous pGT flies from the BG00842, BG01140, BG01322, BG02759 and BG02836 lines exhibited high mortality levels under our experimental conditions, and the BG02142, BG01610, BG02810 and BG02820 lines were homozygous lethal. Thus although all the pGT lines are described as homozygous viable in the database, we found many were not, a feature also reported by Harbison et al [14].

**Figure 2:**
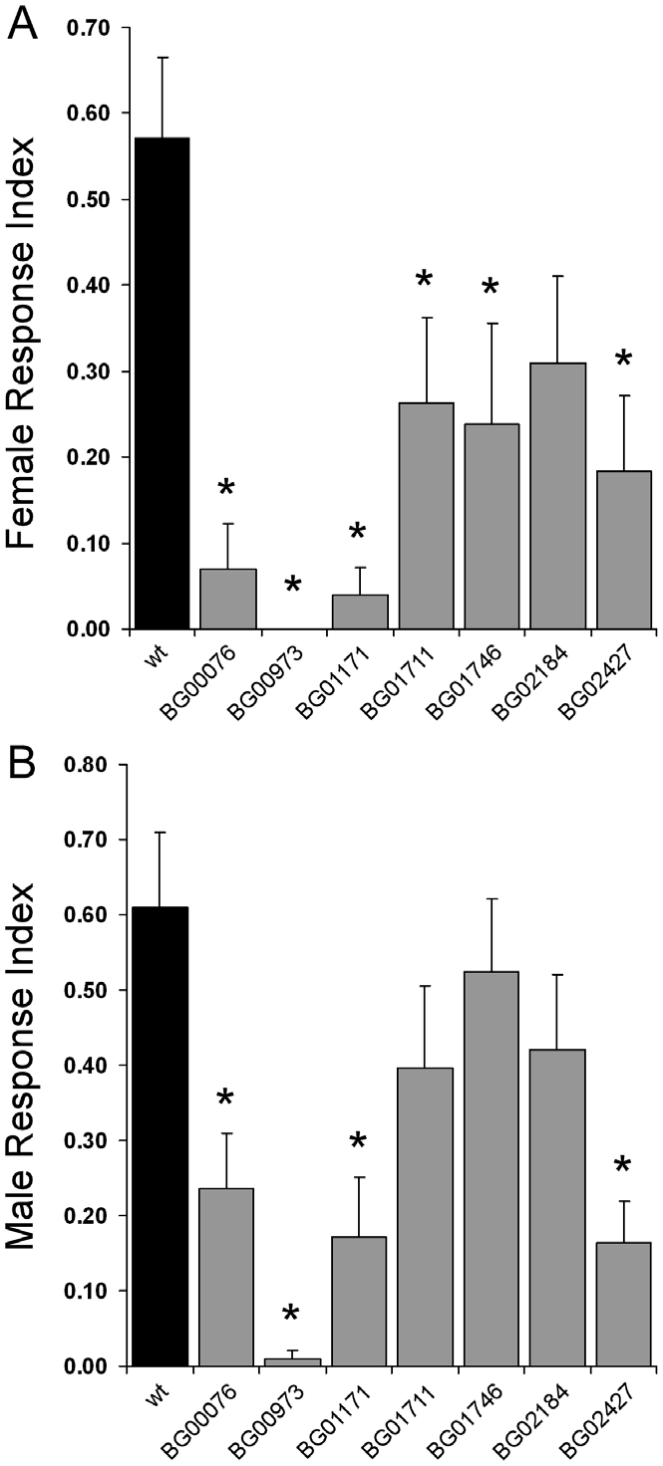
Olfactory Trap Response Index of pGT lines. Defects in olfactory behaviour were tested using an olfactory trap assay. Only seven pGT lines could be tested reproducibly for olfactory trap behaviour because of high mortality rates. The response index (RI) of flies entering traps was recorded at 20-hour intervals over 60 hours and the average at 60 hours is shown. A. Females. B. Males. The pGT lines are represented in numerical order. The error bars represent SEM; n = 10 for all lines. * p<0.05 t-test.

For the remaining seven lines we tested their olfactory-driven behaviour in an olfactory trap assay adapted from Woodard et al [15], using as the attractant a common complex odour, fly food. We found that in four of the lines both female and male flies showed olfactory behaviour deficits compared to the wild type control, and for another two lines only females showed defects (Figure 2). Females of six of the lines; BG00076, BG00973, BG01171, BG01711, BG01746 and BG02427, have a greatly reduced olfactory trap response index (RI) compared to the wild type control (Figure 2A). The male olfactory trap data shows significant differences in RI compared to the wild type control for four of the lines; BG00076, BG00973, BG01171, and BG02427 (Figure 2B). These olfactory behaviour defects are not due to the genetic background of the pGT lines as we tested three other pGT lines that showed no expression in the olfactory organs for olfactory behaviour response and did not observe any differences to the wild type control (Figure S3).

**Figure 3:**
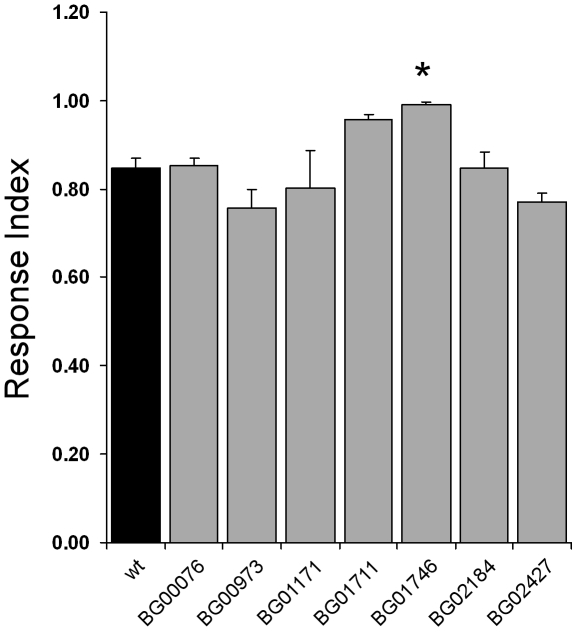
Geotaxis Response Index is normal in pGT lines. Negative geotactic ability was tested to investigate CNS and locomotor function of the lines that exhibited abnormal olfactory behaviour. All lines tested showed negative geotactic behaviour to at least control levels, with one line (BG01746) showing a small increase (* ANOVA, t-test, p<0.007). The pGT lines are represented in numerical order. The error bars represent SEM; n = 5–19.

In many of the lines GFP expression was also observed in the brain and other sensory organs, including neurons in the legs, thus it was possible that failure to enter the traps could be a result of CNS or locomotor defects rather than olfactory defects. A startle-induced negative geotaxis assay was therefore performed to test for such deficits. When tapped/bumped to the bottom of a vial, flies will then quickly climb upward against the force of gravity, a behaviour defined as startle-induced negative geotaxis [16], [17]. This behaviour is dependent on both locomotor ability and geotaxis. None of the pGT lines showed a significant defect in negative geotaxis response in this assay indicating there were no major CNS or locomotor defects in these lines (Figure 3). One line did, however, show a significantly increased response compared to the wild type control.

In order to determine if the olfactory behaviour defects were due to a defect in peripheral olfactory signal transduction we performed electroantennogram (EAG) analysis to test the seven homozygous viable lines for response to a small set of odours (ethyl acetate, pentyl acetate, benzaldehyde, and methyl salicylate) that many ORN functional classes respond to. No mutant EAG phenotypes were observed for any of the pGT lines with these odours (ANOVA, p>0.05; data not shown).

### For BG00076 the olfactory behaviour defects are caused by the pGT insertion

**Figure 4:**
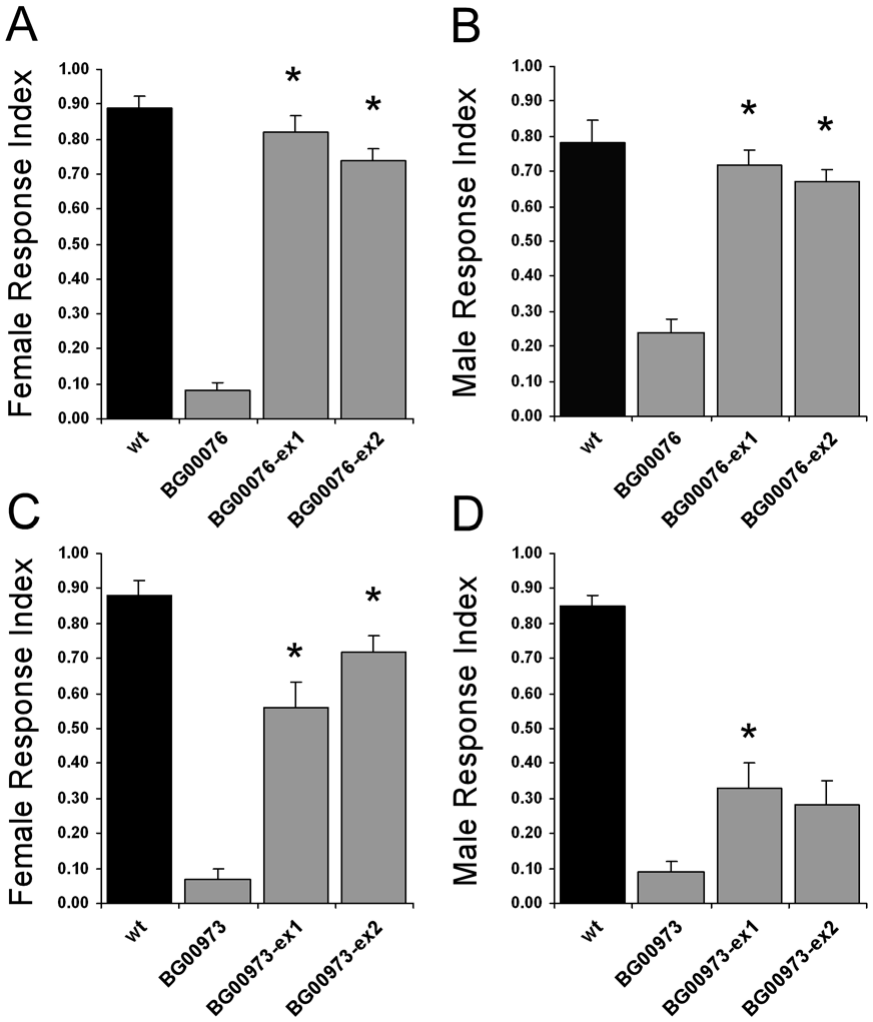
Precise excision of the pGT element in BG00076 and BG00973 restores olfactory behaviour. Comparison of response indices of wild type flies (wt), pGT insertion mutants (BG00076 or BG00973) and two precise excision lines for each (ex1, ex2). Asterisks for excision lines indicate significantly higher responses than pGT mutants (ANOVA, t-test, p<0.01). (A–B) For BG00076 mutant responses are rescued in both ex1 and ex2 in females (A) and males (B). (C–D) For BG00973 mutant responses are rescued in both ex1 and ex2 in females (C) but only in ex1 in males (D). The error bars represent SEM; n = 10 for all lines.

Two of the lines that showed strong behavioural defects in both females and males, BG00076 and BG00973, were selected for further analysis. To confirm that the behaviour defects are due to the pGT element insertion in these two lines we carried out genetic experiments to generate lines in which precise excisions of the pGT element(s) had occurred. We confirmed the precise excision events by performing PCR with primers flanking the insertion sites in *SKIP* and *CG3217* respectively, and sequencing the PCR products. For BG00076 we found that precise excision of the pGT element in two independent excision lines rescued the olfactory response in both females and males (Figure 4A and 4B), confirming that the olfactory behaviour defect is due to the pGT element insertion. For BG00973 precise excision of both of the pGT element insertions (confirmed by PCR experiments for each locus) restored behaviour in females (Figure 4C). In males there was an increased response in the two excision lines, but this was significantly different to the homozygous pGT flies only for one of the excision lines (Figure 4D). Thus further experiments will be required to confirm that the behaviour defects in BG00973 are due to the insertion in *CG3217*.

**Figure 5:**
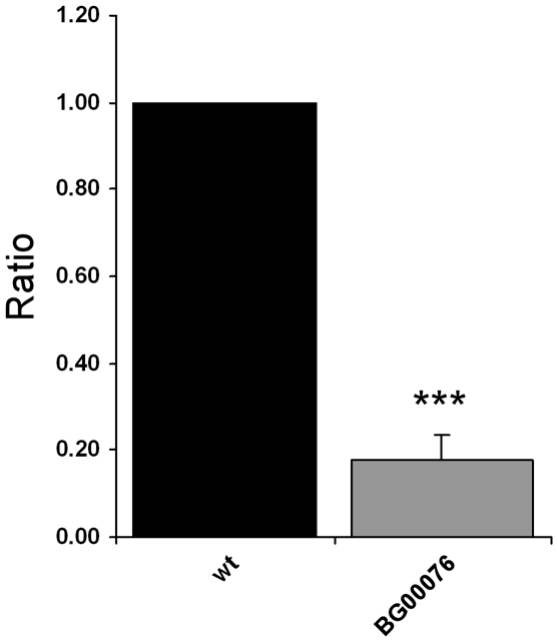
The pGT insert in BG00076 decreases *Skip* expression level. Quantitative real-time PCR analysis of *Skip* expression in whole adult flies. The amount of *Skip* mRNA from CS5 and BG00076 flies was normalized to cyclin K and is indicated in arbitrary units. Values are shown as the mean ± the SEM and are averaged from four separate biological experiments (each replicated in quadruplicate). There is an approximately five fold decrease of *Skip* transcription in line BG00076. *** p<0.001 t-test.

As we had confirmed the pGT insertion was causing the olfactory behaviour defect in BG00076 we performed an experiment to assess the effect of the insertion on expression of the candidate gene, *SKIP*. The expression levels of the *SKIP* gene in control and BG00076 flies were compared using quantitative real-time PCR. This experiment showed that the level of *SKIP* transcript is significantly lower in BG00076 flies than in wild type control flies, with an approximately five fold decrease (normalised to cyclin K) in transcript level (Figure 5).

We were also interested in the fact that, unlike the majority of the lines, BG00076 showed expression in the third antennal segment but not in the second olfactory organ, the maxillary palp. We thus confirmed this finding by performing RT-PCR experiments to determine the tissues in which the *SKIP* gene was expressed. These experiments showed that the *SKIP* gene was expressed endogenously in antennae, head, and body but not in four separate preparations of maxillary palp tissue (Figure S4).

## Discussion

Genetic screens are extremely useful for identifying genes involved in a biological process of interest in an unbiased manner. Various subsets of the collection of pGT lines have previously been screened by others for defects in a number of behavioural phenotypes or morphological traits, such as aggressive behaviour, starvation resistance, sleep patterns, bristle number, and odour guided behaviour [14], [18]–[22]. However, to our knowledge this study is the first to screen them based on expression patterns, followed secondly by analysis of effects on behaviour. An advantage of screening for expression rather than for mutant phenotype is that genes can be identified that may act redundantly such that they do not give mutant phenotypes, and also those that have additional roles outside the olfactory system, such that null mutants are homozygous lethal.

From the screened set of 505 gene trap lines we identified 16 with expression in adult olfactory organs. In 12 of these lines expression of the trapped gene was neuronal, in three it was in accessory cells, and in one line expression was in both cell types. We did not find clear evidence for expression of any of the genes in particular morphological types of sensilla, however in some lines expression may not be in all neurons or all accessory cells, thus this remains possible. Most of the 16 lines also showed expression in other sensory organs, particularly in gustatory organs. This raises the interesting possibility that many of the trapped genes could be utilised in both olfaction and gustation. As the *Or* genes share an ancestry with the gustatory receptors encoded by the *Gr* gene family [23], they may generate neuronal signals in similar manners. In addition members of the *IR* gene family are expressed in both olfactory and taste neurons [9] and may interact with similar genetic pathways in both. There may also be shared developmental pathways or maintenance functions between olfactory and gustatory neurons in which these genes may act.

Of the 16 olfactory-positive lines we were able to test the olfactory-driven behaviour of seven. When the pGT lines were generated they were reported to be homozygous viable [10], [11]. However, nine of the 16 olfactory-positive lines were either homozygous lethal or homozygotes were very unhealthy under our experimental conditions. Other researchers have also reported problems with lethality [14] indicating that many of the pGT insertions are affecting essential genes. This fits with our findings that all but one of the lines are expressed in other adult tissues. Many of the genes we identified encode multiple isoforms, and it is possible that olfactory-specific isoforms exist. Alternatively common isoforms may have pleiotropic functions.

Six of the seven lines we tested using an attractive olfactory trap assay had clear olfactory-driven behaviour defects. For one of the lines with a severe defect we showed that we could rescue olfactory behaviour by precisely excising the pGT element, confirming the olfactory defect is due to the pGT insertion in the *SKIP* gene. For a second line we obtained rescue in females and in one of the two excision lines in males, thus a role for the trapped gene, *CG3217*, in olfaction is possible but further experiments are required to confirm this.

Although the trapped genes in these seven lines are expressed in antennae and six of them affected olfactory behaviour, none of the seven tested showed any defects in the EAG response. There are a number of reasons why this may be the case. The EAG is a summation of receptor potentials and only reflects the primary transduction event, *i.e.* ligand-binding and channel activation, as mediated by the receptor proteins themselves. Other essential functions of olfactory receptor neurons such as action potential generation, firing dynamics, axonal conductivity and targeting, synaptic transmission, and integration of synaptic feedback, are not recorded. In addition, we only tested a limited set of odorants and it remains possible that receptor potentials in neurons responding to other odorants are in fact affected. Most of the trapped genes show expression in the brain, particularly the mushroom bodies, and may affect olfactory behaviour through central rather than peripheral neurons. Finally, the genes we identified may function in olfactory system development or maintenance.

Of the trapped genes we identified, none are obvious candidates for primary events in odour binding and signal transduction. We did not find any *Or*, *IR*, *OBP* genes or proteins that appear likely to directly interact with these. For four of the lines there is more than one candidate gene due to either the presence of two inserts or to differences between the molecular analysis conducted by us and BDGP. Of the twelve lines for which we have only one candidate gene, four of the genes (*shep*, *sbb*, *cpo* and *tai*) encode DNA or RNA binding proteins that have known roles in development, and all these lines showed expression in neurons of the PNS as well as CNS. It is possible that these genes have a role in the development of olfactory sensilla, however they may also affect the more general development of neuronal wiring. For instance *Scribbler (sbb)*, is involved in axon target recognition, axon guidance and larval turning behaviour [24]. *Couch potato* (*cpo*) is known to be involved in PNS development as well as in synaptic transmission [25], [26]. Partial loss of function mutations in *cpo* cause a variety of behavioural phenotypes including abnormal phototaxis, geotaxis, flight ability and ether recovery [25]. We were unable to test for an olfactory behaviour defect as the line that trapped *cpo*, BG02427, is homozygous lethal. However, a different pGT insertion in this gene was found to affect olfactory behaviour in a screen for avoidance of benzaldehyde [21]. This line was not in our screened set as it is no longer available, and presumably it is a partial loss of function mutation as it was able to be phenotypically tested in their study.

Some of the other trapped genes may be involved in neuronal signalling or function. Two of the genes we identified that are expressed in olfactory neurons encode serine/threonine kinases, proteins well known to perform roles in regulation of cell signalling pathways. Both of them are likely to perform many essential roles not related to olfaction and we were unable to test these lines with behaviour or EAGs as they were homozygous lethal or unhealthy. *Pka-C1* (line BG02142) is of interest as it encodes a catalytic subunit of a cAMP-dependent kinase (PKC) that is well known to play a role in mid-term memory formation. Hypomorphic mutations in this gene show memory defects in an olfactory learning paradigm [27], [28] and this has been linked to expression in the mushroom bodies of the brain. Here we show it is also expressed in ORNs and may thus also play a role in these peripheral neurons. Although it appears the Or proteins primarily function as directly ligand-gated ion channels, there is also evidence that Or activation generates cAMP [8] and that cAMP can activate the Orco receptor via PKC-induced phosphorylation [29]. Thus *Pka-C1* may play a role in modulating Or signalling. We were unable to test this line for behaviour or EAG defects as it is homozygous lethal. The second kinase, *Pdk1* (line BG02759), seems less likely to play a specific role in olfactory signalling or processing as it is involved in regulation of signalling pathways that regulate cell growth, size and apoptosis [30] and thus may have developmental roles. Elucidating the role of both these genes in the olfactory system will require methods such as generation of mosaic clones or tissue-specific RNAi experiments.

A third gene of interest that was expressed in neurons is the *SKIP* (Shal K^+^ channel interacting protein) gene (line BG00076). This gene is so named as one of its isoforms, SKIP3, was found to interact with the cytoplasmic C terminal domain of the voltage-gated potassium channel Shal1 [31]. In cell culture experiments SKIP3 was shown to inhibit the fast inactivation of Shal1 currents in some but not all neurons [31]. The many different voltage gated K^+^ channels contribute to the diversity in electrical properties of neurons [32] and changes in specific Na^+^/K^+^ conductances can modify action potential firing rates and dynamics in ORNs [33]. *SKIP* expression was observed throughout the CNS and PNS in *Drosophila* embryos [31]. In the gene trap line we observed expression in the brain, antennal second segment, legs and wings. However, we did not see expression in the maxillary palps, which carry both olfactory and mechanosensory neurons, nor in the labellum, which has taste neurons. This may mean that the BG00076 insertion traps an isoform that may have a more specific role. For this line we confirmed that expression levels of the *SKIP* gene are lowered due to the pGT insertion and that precise excision completely rescues the olfactory behaviour phenotype. There was no effect on the EAG, but a limited set of odours was tested, and in addition if the gene is involved in synaptic transmission then we would not expect to see a primary EAG defect.

The BG00973 line was the only one that was neuronally expressed but did not show expression in the brain, and may therefore be specific to peripheral neurons. In our behavioural experiments this line showed the strongest olfactory phenotype. The candidate gene is a gene of unknown function, *CG3217* or *CkIIα-i3*. Based on yeast 2-hybrid experiments, this gene has been suggested to encode a protein that interacts with a casein kinase II alpha subunit and is thus called *CkIIα-i3* [34]. However, whether this is reflective of an *in vivo* interaction remains to be verified. The CG3217 protein shows homology to the human actin-bundling protein TRIOBP, which contributes to the rigidity of stereocilia in hair cells, and mutations in this gene cause deafness [35]. This is more suggestive of a role in neuron architecture rather than in signalling.

A final example of a gene that may contribute to neuronal function is the *Mctp* gene (line BG01140), which interestingly was not expressed in neurons but accessory cells. Although expression in this line appeared olfactory-specific in adult flies, the line was homozygous lethal and thus the trapped gene must have an essential function. The *Mctp* gene encodes a *Drosophila* homologue of mammalian MCTP proteins, proteins of unknown function that contain both transmembrane domains and multiple C_2_ domains [36]. C_2_ domains are Ca^2+^-binding modules that appear to be important for Ca^2+^ sensing and regulatory functions rather than for Ca^2+^ buffering. They are usually found in proteins involved in signal transduction or in membrane trafficking, for example phospholipases, protein kinase C and synaptotagmins. Due to their transmembrane domains, MCTP proteins are proposed to play roles in Ca^2+^ signalling at the membrane [36]. The accessory cells secrete many proteins into the sensillum lymph that surrounds the ORN dendrites that are critical for ORN function, the *Mctp* gene may play a role in this process.

### Conclusion

While detailed studies have been performed over the past decade to identify large families of receptor genes and study their role in *Drosophila* olfaction, relatively few other genes involved in olfactory system function and development have been identified. This study has identified a set of 16 genes that are expressed in the adult *Drosophila* olfactory organs, mostly in neurons but in several cases in accessory cells. The genes include some known genes with roles in axon guidance and peripheral nervous system development, but we also uncovered interesting new genes that have not been well characterised and have potential roles in olfactory signalling, higher order information processing, or olfactory system maintenance or development. As most or all of the genes have expression and thus likely roles outside the olfactory system, further characterisation of their roles in olfaction will require tissue-specific loss of function experiments.

## Materials and Methods

### *Drosophila* stocks

All flies were reared on yeasted semolina/syrup medium in 40 ml vials at 22°C and normal daylight. 505 P{GT1}/pGT strains, constructed as part of the Berkeley *Drosophila* Gene Disruption Project [10], [37] were obtained from Bloomington Stock Centre, as was the UAS-mcd8:GFP reporter line (stock BL5137). Canton-S flies were used as the control line for phenotypic assays and molecular experiments as this is the genetic background of the pGT lines.

### Generation and verification of precise excision lines

Precise excisions of pGT elements were generated using standard crossing schemes introducing the Δ2–3 transposase to re-mobilize the P-element. Excision events were identified by PCR with primers designed to sequences flanking the insertion sites. PCR products were sequenced to verify the precise excisions.

### GFP screen for olfactory expression patterns

Virgin homozygous UAS-mcd8GFP females were collected en masse and crossed to homozygous or heterozygous males from the pGT lines. The heads of 3–10 day old adult F1 progeny were removed to facilitate screening of the olfactory organs. This was performed on a microscope slide in 1× PBS and heads were examined for GFP expression on a Leica DMLB compound microscope under fluorescent light (I3 filter). For the 16 olfactory-positive lines further experiments were performed to examine GFP fluorescence in other tissues. GFP fluorescence was assessed in legs, wings, mouthparts and heads by examining either heads or whole flies under both a stereomicroscope and the compound microscope. GFP fluorescence in the brain was assessed from the cryosections generated for determining cellular localisation of the GFP signal, described below.

### Immunohistochemistry

Heads of 3–10 day old adults were embedded in Tissue-Tek and frozen on dry ice. Frontal sections were cut at 10 µm using a Leica cryostat set at −20°C and applied to Poly-L-lysine treated slides. Sections were fixed in 4% paraformaldehyde for 30 minutes at room temperature (RT), then washed three times in PBS-Tx (PBS+1% Triton-X) for 5–10 minutes and blocked in PBS-Tx/1% BSA for either 1–2 hours at RT or overnight at 4°C. Slides were removed from blocking solution and ∼200 µl of primary antibody applied under a bridged coverslip and incubated overnight at 4°C. The primary antibodies were diluted in PBS-Tx/1% BSA; anti-elav (mouse, DSHB) was used at 1/10–1/50 and anti-GFP (rabbit, Clontech) was used at 1/50. Secondary antibodies; Alexa anti-mouse 594 and Alexa anti-rabbit 488 (Molecular Probes), were diluted to 1/250 with PBS-T/1% BSA. Sections were viewed using a Leica DMLB with ebq 100-isolated light source and I3, and N2.1 filters for green and red fluorescence respectively.

### Rapid amplification of cDNA ends (3′ RACE)

Head and antennal cDNA samples were prepared from pGT line flies and first strand synthesis performed with an adapter-dT primer, QT (CCAGTGAGCAGAGTGACGA GGACTCGAGCTCAAGC T(17)), which contained the sequence of the adapter primer required for the subsequent nested PCR amplification. PCR amplification of cDNA ends was performed using a pGT vector specific forward primer, *white-1* (ATTCTCATCGTGAGCTTCCGGG) and the adapter primer QO (CCAGTGAGCAGAGTGACGAG). Products were run on a 1% agarose gel and DNA bands purified and sub-cloned into the pGEM-T Easy vector (Promega) for sequencing. Sequences obtained were used in BLAST searches of the *D. melanogaster* genome database to identify the exons of genes that had spliced to the *mini-white* gene of the pGT vector.

### Quantitative PCR

Whole fly mRNA samples were prepared from 40 flies using the Qiagen RNeasy kit (per manufacturers instructions) and mRNA was eluted in 30–40 µl of RNase free H_2_O. mRNA was quantified and DNase treated and 4 µg used for reverse transcription with random primer mix. Real-time PCR was performed using a Rotor Gene 6000 with a 72 well rotor (Corbett Research). Primers for real-time PCR were designed using Perlprimer software available at http://perlprimer.sourceforge.net. Real-time PCR reactions consisted of: 10 µl 2× Sensimix, 1.6 µl 5 µM of each primer, 0.5 µl 50× SYBR, 2 µl of a 1/5 dilution of cDNA in a final volume of 20 µl. Reaction conditions used were the default parameters (SYBR® green I) but with the run extended to 55 cycles and the gain set at 10 for the SYBR green channel. Four biological replicates were performed in quadruplicate and the amount of gene product in each sample was determined using the comparative quantitation method using the Rotor Gene 6.0 software (Corbett Research) normalised to two internal controls Cyclin K and RPL32.

### Behavioural Assays

#### Olfactory Trap assay

Flies 5–7 days post-eclosion were tested using the olfactory trap method of Woodard et al [15]. The attractant used was ∼400 µl of fly food medium placed in the lid. As a trap entry hole that is big enough for females to enter will allow males to escape due to their smaller body size, males and females were tested separately with yellow pipette tips cut off at different levels to adjust the trap entry point (0.8 cm from end for males and 0.9 cm from end for females. The completed trap apparatus was placed in a petri dish layered with 1% agarose to provide moisture and the petri dish wrapped with parafilm to prevent moisture loss. Experiments were run for 60 hours in the dark. The number of trapped flies was counted at 20, 40 and 60 hours. A response index (RI) was calculated where the number of flies trapped was divided by the total and averaged over the number of tests (±S.E.M.s). Data was analysed statistically using one way analysis of variance (ANOVA) and unpaired t-tests to compare each line to Canton-S.

#### Negative Geotaxis assay

Negative geotactic ability was measured to test all lines for normal central nervous system (CNS) function and indirectly locomotive function. Tests were conducted at the same time of day (afternoon) to exclude the effect of bi-modal peaks in locomotor activity. Groups of 10–15 individuals were placed in a 110×25 mm vial marked with a line drawn horizontally 8 cm from the bottom. Flies were aspirated into the vial and given 30 seconds to recover. The flies were bumped to the bottom and given 10 seconds to display startle-induced negative geotaxis by migrating to the top of the vial against the force of gravity [16], [17]. After 10 seconds the number of flies above the 8 cm line was recorded. To account for differences in the force of the bump, the same group of flies were tested with 5 bumps and an average of this taken as one replicate. A response index (RI) was calculated where the number of flies above the line is divided by the total and averaged over 5–10 tests (±S.E.M.s). Data was analysed statistically using one way ANOVA and unpaired t-tests to compare each line to Canton-S controls.

### *In situ* hybridisations to *Drosophila* polytene chromosomes

Salivary glands were extracted from wandering 3^rd^ instar larvae of the pGT lines. Polytene chromosome squashes and *in situ* hybridisation with a DIG-labelled *Gal4* probe were performed using the methods described in [38].

### Reverse Transcriptase PCR

Extraction of RNA from small tissue samples such as antennae and palps or small quantities of *Drosophila* heads or bodies was performed using a guanidine thiocyanate method [3]. First strand cDNA synthesis was performed using the Invitrogen Superscript III kit with 1 µl oligo-dT as per the manufacturer's instructions. PCR reactions were performed in a 50 µl volume and consisted of: 1× Taq buffer, 0.4 mM dNTP mixture, 2.5 mM MgCl_2_, 1 µM of each primer, 1 µl template DNA (1–2 µg genomic DNA) and 1 unit of *Taq* polymerase. Reactions were performed in a Hybaid PCR express from Integrated Sciences using standard cycling conditions. Primers were designed to flank introns such that cDNA products could be clearly distinguished from any contaminating genomic DNA.

### Southern blots

A DNA fragment corresponding to the *Gal4* gene was PCR amplified and 2 µl of purified (Gene Clean) product labelled with [α^32^P] dATP by nick translation according to the DecaPrime II protocol (Ambion, Cat #1455 from Geneworks). All hybridisations were performed with a low stringency hybridisation buffer (30% formamide, 5× SSPE, 5× Denhardt's solution, 100 µg/mL herring sperm DNA, 0.1% SDS) at hybridisation temperatures of 55–60°C. Nylon filters (Hybond-N+) were incubated in hybridisation tubes with 10 mL of pre-hybridisation solution for 2 hours in a rotor oven. The probe was boiled for 10 min and placed on ice for 5 min, before being added to the existing pre-hybridisation solution. Hybridisation was performed for 16–20 hours. Filters were washed the next day at very low stringency (Wash 1, 10× SSC, 0.1% SDS) for 20 mins to remove excess probe, then at medium stringency (1× SSC, 0.1% SDS) twice for 30 min. Filters were placed in hypercassettes with auto-radiographic film at −70°C for 1–5 days, depending on signal intensity.

## Supporting Information

Figure S1 **Southern blots show multiple insertions in some pGT lines.** 5 µg of genomic DNA from each pGT line was digested with EcoR1 and HindIII and probed with *Gal4* DNA. A. Southern blot of all 16 lines. Most lines showed a single band, however some appeared to have multiple bands. For these a second blot was performed where the gel was run for a much longer time period to achieve better band separation. In this case three lines were confirmed to have more than one band (B). BG01171 and BG00973 have two bands indicating two inserts. BG02820 has three bands indicating three inserts. No bands were seen in a wild type negative control. Southern blots were exposed for 2 days.
(TIF)

Figure S2 **Larval polytene chromosome** ***in situ*** **hybridizations.** Larval polytene chromosome squash*es* were probed with DIG-labeled *Gal4* probes. A. For line BG01140 one signal was observed at ∼55F on chromosome 2R. B and C. For line BG02836 two signals were observed, one at ∼18D on chromosome X (B) and the second at ∼67E on chromosome 3L (C).
(TIF)

Figure S3 **Olfactory behaviour defects are not due to the genetic background of the pGT lines.** Comparison of the olfactory trap response index of Canton S to three control pGT lines that are not expressed in the olfactory organs shows no significant differences (ANOVA). A. Females. B. Males. The error bars represent SEM; n = 7 for all lines.
(TIF)

Figure S4 **The** ***SKIP*** **gene is expressed in antennae but not in maxillary palps.** RT-PCR products obtained from Canton S flies. A. *SKIP* expression was observed in body (B), heads from which olfactory organs had been removed (H), and antennae (A) but not in maxillary palps (P). (N) is the negative control with no DNA template. Expected PCR product sizes are 220 bp for cDNA and 600 bp for genomic DNA. These results are representative of four biological replicates. B. To show that the maxillary palp cDNA preparation used in (A) does contain cDNA we also show the RT-PCR results for the *Mctp* gene, which is expressed in both antennae and maxillary palps. Expected PCR product sizes are 395 bp for cDNA and 565 bp for genomic DNA.
(TIF)
