# Supplementary figures and images for: A Screen for Genes Expressed in the Olfactory Organs of Drosophila melanogaster Identifies Genes Involved in Olfactory Behaviour
Source: PLoS One. 2012 Apr 18;7(4):e35641. doi: 10.1371/journal.pone.0035641 (PMC3329464; doi:10.1371/journal.pone.0035641)

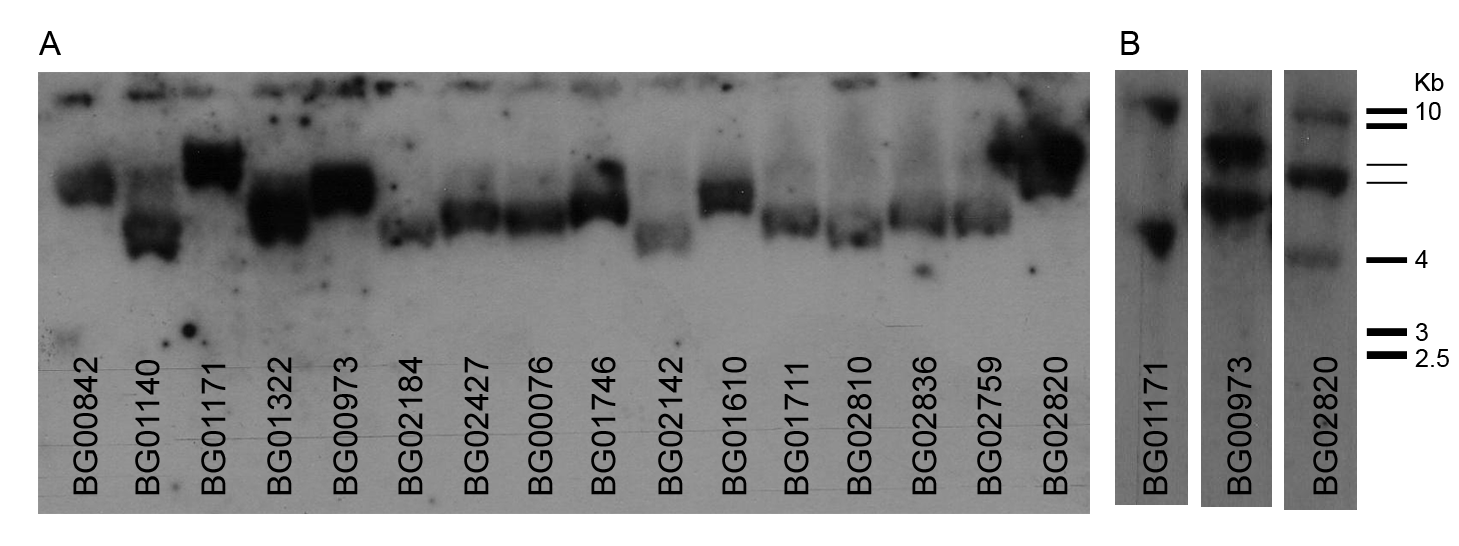

Supplement: Figure S1 — Southern blots show multiple insertions in some pGT lines. 5 µg of genomic DNA from each pGT line was digested with EcoR1 and HindIII and probed with Gal4 DNA. A. Southern blot of all 16 lines. Most lines showed a single band, however some appeared to have multiple bands. For these a second blot was performed where the gel was run for a much longer time period to achieve better band separation. In this case three lines were confirmed to have more than one band (B). BG01171 and BG00973 have two bands indicating two inserts. BG02820 has three bands indicating three inserts. No bands were seen in a wild type negative control. Southern blots were exposed for 2 days. (TIF) [file pone.0035641.s001.tif]

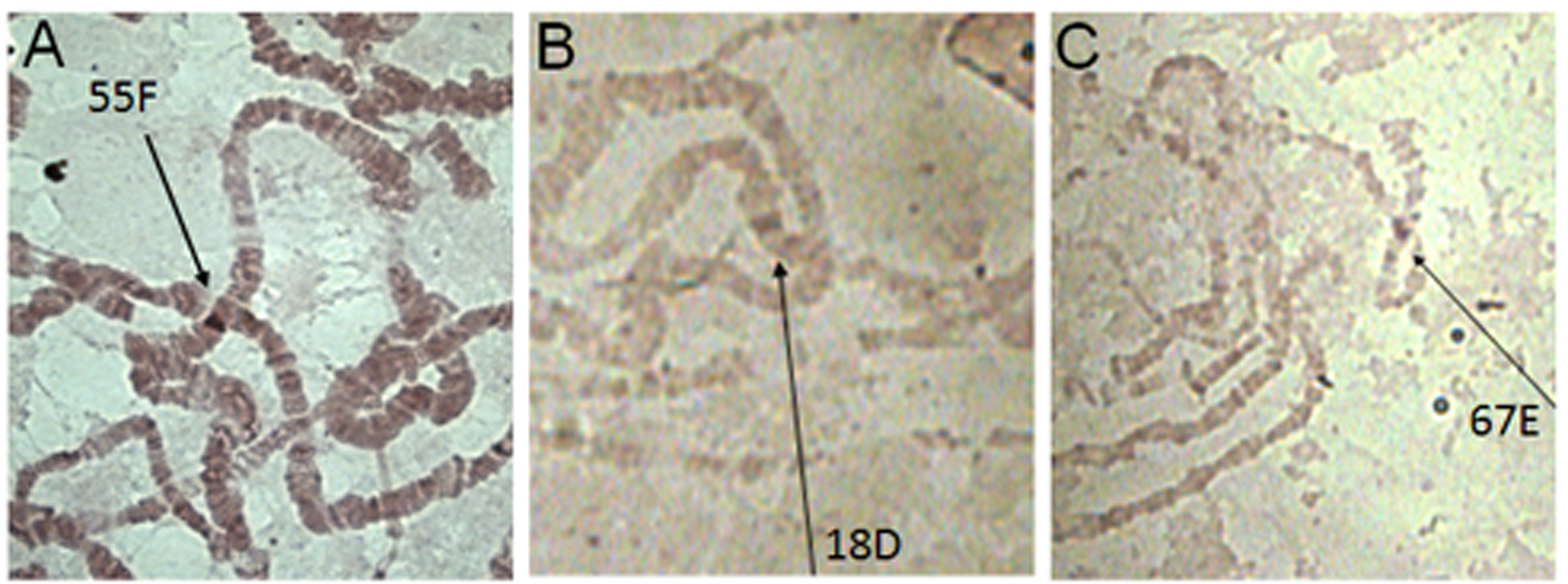

Supplement: Figure S2 — Larval polytene chromosome in situ hybridizations. Larval polytene chromosome squashes were probed with DIG-labeled Gal4 probes. A. For line BG01140 one signal was observed at ∼55F on chromosome 2R. B and C. For line BG02836 two signals were observed, one at ∼18D on chromosome X (B) and the second at ∼67E on chromosome 3L (C). (TIF) [file pone.0035641.s002.tif]

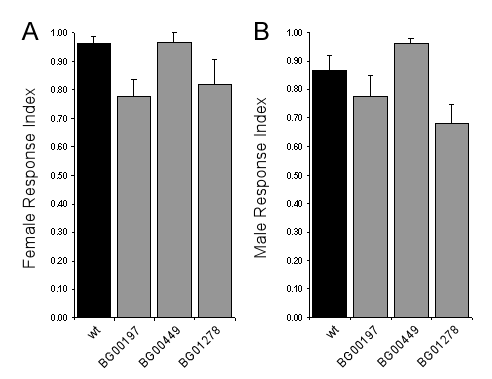

Supplement: Figure S3 — Olfactory behaviour defects are not due to the genetic background of the pGT lines. Comparison of the olfactory trap response index of Canton S to three control pGT lines that are not expressed in the olfactory organs shows no significant differences (ANOVA). A. Females. B. Males. The error bars represent SEM; n = 7 for all lines. (TIF) [file pone.0035641.s003.tif]

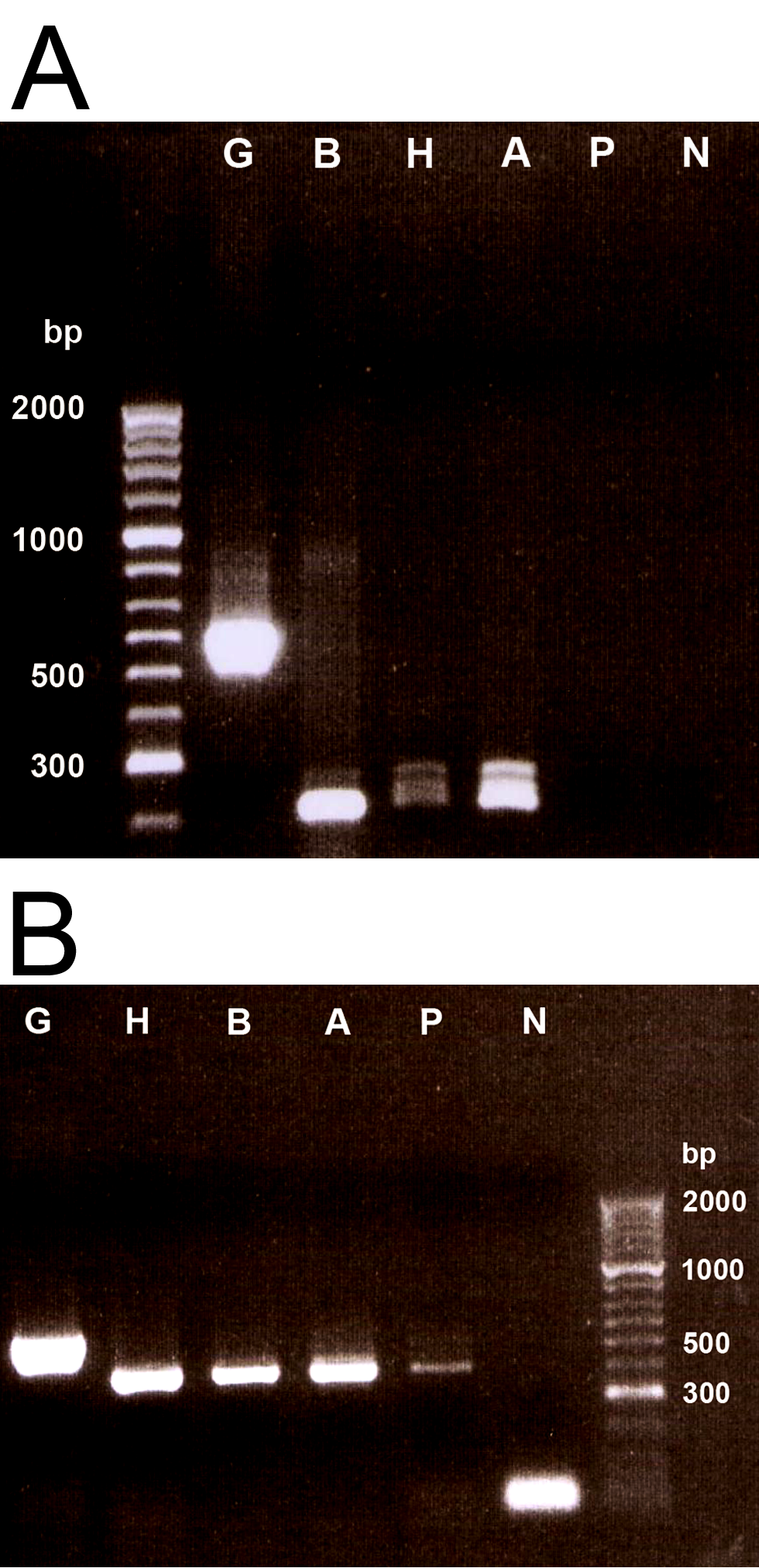

Supplement: Figure S4 — The SKIP gene is expressed in antennae but not in maxillary palps. RT-PCR products obtained from Canton S flies. A. SKIP expression was observed in body (B), heads from which olfactory organs had been removed (H), and antennae (A) but not in maxillary palps (P). (N) is the negative control with no DNA template. Expected PCR product sizes are 220 bp for cDNA and 600 bp for genomic DNA. These results are representative of four biological replicates. B. To show that the maxillary palp cDNA preparation used in (A) does contain cDNA we also show the RT-PCR results for the Mctp gene, which is expressed in both antennae and maxillary palps. Expected PCR product sizes are 395 bp for cDNA and 565 bp for genomic DNA. (TIF) [file pone.0035641.s004.tif]
